# WPL-Based Constraint for 3D Human Pose Estimation from a Single Depth Image

**DOI:** 10.3390/s22239040

**Published:** 2022-11-22

**Authors:** Huiqin Xing, Jianyu Yang

**Affiliations:** School of Rail Transportation, Soochow University, 8 Jixue Road, Xiangcheng District, Suzhou 215131, China

**Keywords:** WPL-based constraint, human-tree, distal and proximal joints

## Abstract

Three-dimensional human pose estimation from depth maps is a fast-growing research area in computer vision. The distal joints of the human body are more flexible than the proximal joints, making it more difficult to estimate the distal joints. However, most existing methods ignore the difference between the distal joints and proximal joints. Moreover, the distal joint can be constrained by the proximal joint on the same kinematic chain. In our work, we model the human skeleton as the tree structure called the human-tree. Then, motivated by the WPL (weighted path length) in the data structure, we propose a WPL-based loss function to constrain the distal joints with the proximal joints in a global-to-local manner. Extensive experiments on benchmarks demonstrate that our method can effectively improve the performance of the distal joints.

## 1. Introduction

Three-dimensional human pose estimation from a single depth image is a fast-growing research area and has drawn long-standing attention in the past decades. It has wide applications in the computer vision field, such as robotics and human–computer interaction [1,2,3,4]. Recently, deep-network-based methods [5,6,7,8,9,10,11,12,13,14,15,16,17,18,19,20] have achieved promising results in 3D human pose estimation. However, it is still a challenging task because the human body is highly deformable and suffers from frequent self-occlusions.

The 3D human pose estimation method can be divided into generative methods [15,17,20] and discriminative methods [5,11,13,21,22,23,24,25,26,27,28,29,30]. The generative methods estimate the 3D human pose by learning the correspondence between the pre-defined human body model and the input depth image. Even though these methods can introduce prior knowledge of the human body into models, the process of fitting the complicated human model to the depth image is time costly. The discriminative methods use the pre-trained body part detectors to estimate each joint from the input depth image. These methods do not need the pre-defined human body template and can directly regress the positions of joints. In this work, we adopt the discriminative method for 3D human pose estimation.

Conventional discriminative methods mainly rely on random forest, such as hough forests [21], random ferns [22], and random tree walks [23]. Recently, CNN-based discriminative methods have achieved promising results in 3D human pose estimation. Haque et al. [28] learned viewpoint-invariant features using CNN for 3D human pose estimation, which makes the model more robust to viewpoint variations. Moon et al. [26] used the 3D voxelized depth map as input and 3D CNN for human pose estimation. However, due to the numerous parameters, the training process is challenging. Kim et al. [11] proposed projecting the depth data in various directions to fully use the depth information. Xiong et al. [27] proposed the use of anchors to simultaneously estimate human joints from different viewpoints. However, the spatial information of the human body is ignored. Though the above methods can effectively improve the average accuracy of human joints, we find that the accuracy of the distal joint (such as the hand and foot) is lower than other human joints. Compared with other joints, the distal joints are more flexible. Therefore, the distal joints are more difficult to estimate. In this paper, we are devoted to alleviating the situation and improving the performance of 3D human pose estimation.

**Figure 1:**
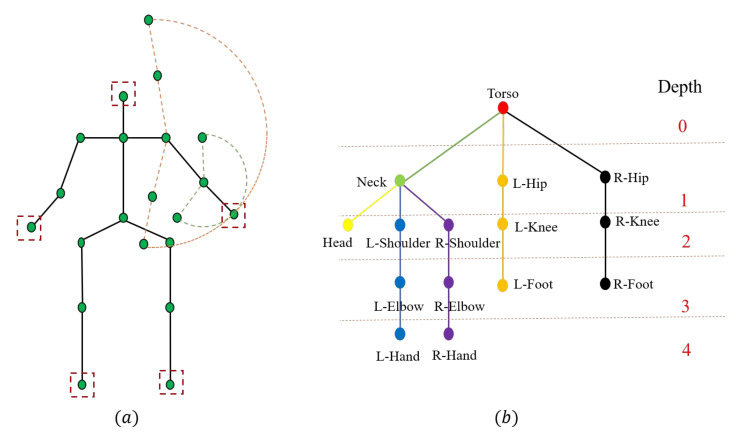
(**a**) The structure of the human skeleton model. The distal joints are denoted in the red dotted box. The active range of the right hand is denoted by the orange dotted line when the position of the right elbow is unknown. The active range of the left hand is denoted by the green dotted line when the position of the right elbow is known. (**b**) The structure of the human-tree model. The depth of the joint is denoted on the right side.

As we all know, the muscle group surrounding the torso is one of the core muscle groups of the human body [31]. Most human movements are performed under the control of the muscles surrounding the torso. Moreover, the torso is a crucial joint connecting the upper and lower limbs, as shown in Figure 1a. We can access any joint from the torso through several bones and joints. These multiple bones and joints are usually combined and named the kinematic chain. For a specific kinematic chain, we divide joints into proximal joints and distal joints according to their distance from the human body. For example, on the kinematic chain of the right upper limb, the right hand is the distal joint, and the right shoulder is the proximal joint. In the open kinematic chain, the active range of the distal joint when the position of the proximal joint is unknown is larger than that of the proximal joint. If we know the position of the proximal joint, the active range of the distal joint will be further constrained on the same kinematic chain. In other words, the proximal joint can constrain the position of the distal joint. If we can make use of this constraint, the accuracy of the distal joint can be effectively increased.

We note that there is a data structure named tree, where the leaf node can be reached from the root node through several intermediate nodes [32]. Inspired by this, we also model the human skeleton as the tree structure called human-tree, as shown in Figure 1b. In the human-tree, the torso is considered the root node, and the distal joints are considered the leaf nodes. Each branch of the human-tree represents a kinematic chain and the connections between two nodes represent the bones. In the tree structure, each leaf node contains a built-in feature called the weight path length (WPL), which is the product of the weight of the leaf node and the path from itself to the root node [32]. This allows us to use the WPL of the proposed human-tree to constrain the distal joints. Specifically, we define the weight of the distal joint according to the sum of the bone lengths between itself and the torso joint, and the path from itself to the torso joint is equal to the number of bones between the two joints. Then, we calculate the loss between the estimated and ground-truth human-tree, called global loss. Of course, not only can the torso constrain the distal joint, but other proximal joints can also constrain the distal joint. The kinematic chains from other proximal joints to the distal joint can be viewed as the subtrees of the human-tree. Similarly, we calculate the loss between the estimated and ground-truth subtree of the human-tree, called local loss. Finally, the local loss and global loss are combined to train the parameters in the model to constrain the distal joints in a global-to-local manner.

The effectiveness of our proposed method is validated on two human body datasets (ITOP-side [28] and ITOP-top [28]). Extensive experimental evaluation and empirical analysis are provided, as well. The main contributions of this paper are as follows:

- We propose a WPL-based loss function for 3D human pose estimation, which can improve the accuracy of the distal human joints effectively.
- The proposed WPL-based function can constrain the estimated human pose in a global-to-local way.
- Extensive experiments demonstrate that our method outperforms some competitive methods on two human pose datasets.

The rest of this paper is organized as follows. In Section 2, we introduce the related work of 3D human pose estimation. In Section 3, we illustrate the details of the proposed method. The experimental results and discussion are presented in Section 4. Finally, we conclude the paper in Section 5.

## 2. Related Works

There have been many methods for 3D human pose estimation from a single depth image in recent years, which can be mainly classified into two categories: generative methods [15,17,20] and discriminative methods [5,11,13,21,22,23,24,25,26,27,28]. In this section, we briefly show the most relevant works of 3D human pose estimation. In addition, since we are devoted to constraining the human pose with spatial information, we also discuss the related work of spatial constraints in human pose estimation.

*Generative methods.* Generative methods first use the extracted features to estimate the 2D coordinates of joints, then infer the 3D poses from the 2D coordinates. Martinez et al. [15] used a simple and fast feed-forward network (FFN) to tackle the 2D-to-3D human pose estimation task. Wang et al. [33] first generated the heatmap of each joint using the FCN, then inferred the human pose using the existing MatchNet [34]. Zhang et al. [20] first estimated the 2D human pose, then used PointNet [35] to extract the embedded features for the 3D human pose estimation. The performance of these methods on 3D human pose estimation is significantly affected by the accuracy of the estimated 2D pose.

*Discriminative methods.* Discriminative methods are used to directly regress the 3D coordinates of human joints from the input depth image. The conventional discriminative methods mostly rely on random forests. For example, in [5,24], each pixel is classified into different body parts, then the 3D coordinates of joints are estimated via the approach based on Mean Shift. In recent years, CNN-based discriminative methods have achieved promising results on 3D human pose estimation. Haque et al. [28] are devoted to extracting the invariant features in different viewpoints for 3D human pose estimation. In [26], the 3D voxelized depth map is fed into the 3D CNN, and the network estimates the likelihood of each body joint for each voxel. Kim et al. [11] proposed the projection of the depth data in various directions to fully use the depth information. In [36], the cleaned and transformed point set is used to match the pre-defined prototypes, then the 3D human pose is estimated from the improved point set. Marin-Jimenez et al. [13] represented the 3D human pose as the weighted sum of the pre-defined prototypes, and the weights can be learned using ConvNet. Xiong et al. [27] proposed the extraction of features from different viewpoints using the anchors for 3D pose estimation. Although the discriminative methods can directly regress the 3D human pose without the time-costly process of fitting the complicated human model to the depth image, the performance of these methods on self-occlusion [37,38] human poses is poor.

*Spatial constraints for the human pose.* Some spatial constraints are proposed to constrain the human pose to improve the accuracy of the 3D human pose. Ganapathi et al. [39] used the enhanced ICP-based model to introduce the free-space constraints into their model. L He et al. [14] used the graphical model to exploit structural constraints. Shuang L A et al. [40] proposed a structure-aware regression model where the pose is represented by human bones. Ding M et al. [41] proposed the articulated Gaussian kernel correlation to introduce the kinematical chain structure into the model.

We note that the accuracy of the distal joint is lower than other joints in the same sample. As a result, we are committed to improving the performance of the distal joints by employing a novel spatial constraint in our paper.

## 3. Method

### 3.1. Overview

**Figure 2:**
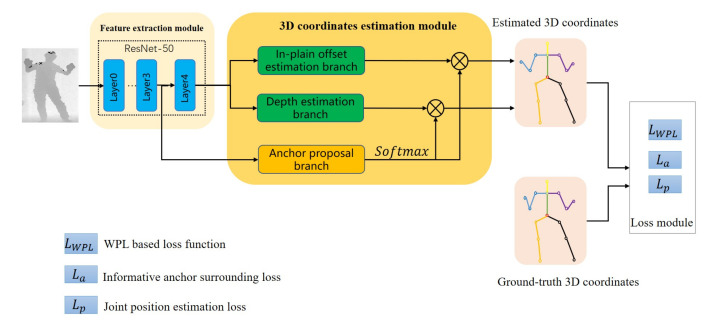
The framework of the proposed method is based on A2J. The feature extraction module first extracts the feature of the input depth image. Then, the 3D coordinates estimation module estimates the 3D positions of joints using the extracted features. Finally, the estimated and the ground-truth 3D coordinates are used to calculate the global-to-local WPL-based loss, informative anchor surrounding loss, and joint position estimation loss.

The framework of our method is shown in Figure 2. The architecture of the network consists of three modules: (1) feature extraction module, (2) 3D coordinates estimation module, and (3) Loss module. The single depth image is first sent to the feature extraction module to extract the feature of the depth image. The extracted features are then fed into the 3D coordinates estimation module to estimate the 3D coordinates of joints. Finally, the estimated and the ground-truth 3D coordinates of joints are sent to the Loss module to calculate the global-to-local WPL-based loss, informative anchor surrounding loss, and joint position estimation loss. Then, the above three losses are backpropagated to update the parameters of the model. In this work, the feature extraction module and 3D coordinates estimation module all refer to the A2J [27] model. Details of the A2J model are described in Section 3.2. This section will introduce the principles of the human-tree model and the WPL-based loss function.

### 3.2. The Framework of A2J

In A2J, the anchors are densely preset on the input depth image to estimate the position of each joint from different viewpoints in an ensemble way. ResNet-50 is used as the feature extraction module to extract the depth map features. The 3D coordinate estimation module is composed of three branches: (1) the in-plain offset estimation branch, (2) depth estimation branch, and (3) anchor proposal branch. The in-plain offset estimation branch and depth estimation branch are used to estimate the 2D coordinates and depths of each joint by all the preset anchors, respectively. The anchor proposal branch is used to estimate the anchor weights. Finally, the 3D coordinates of each joint are acquired by the weighted sum of all the results estimated by the preset anchors. The process of calculating the 3D coordinates is as follows:

(1) $${\hat{S}}_{j}=\sum_{a\in A}{\tilde{P}}_{j}\left(a\right)O_{j}\left(a\right),$$

(2) $${\hat{D}}_{j}=\sum_{a\in A}{\tilde{P}}_{j}\left(a\right)D_{j}\left(a\right),$$

where ${\hat{S}}_{j}$ and ${\hat{D}}_{j}$ represent the estimated 2D coordinates and depth of joint *j*, respectively. A represents the set of the preset anchors, *a* represents the anchor, ${\tilde{P}}_{j}\left(a\right)$ represents the weight of anchor *a* to joint *j*, and $O_{j}\left(a\right)$ and $D_{j}\left(a\right)$ represent the 2D coordinates and depth of joint *j* estimated by anchor *a*, respectively.

However, the spatial relationship between human joints in A2J is ignored when each preset anchor estimates the positions of joints. In our work, we are devoted to making up the weakness of A2J and proposing a new spatial constraint: the proximal joint can constrain the distal joint.

### 3.3. Human-Tree Model

Figure 1a shows the human skeleton model in the ITOP [28] human pose dataset. The human skeleton model is composed of several joints and connections (that is, bones) between joints. Joints on one kinematic chain can be divided into two types: proximal joints and distal joints. The distal joints are denoted in the dotted line box in Figure 1a, which includes the head, the right/left hand, and the right/left foot. As shown in Figure 1a, the possible position range of the right hand joint is denoted by the orange dotted line when the position of the right elbow joint is unknown, and the possible position range of right hand joint is denoted by the green line when the position of the right elbow joint is known. It can be seen if we know the position of the right elbow joint, the possible position range of the right hand joint will be smaller. In other words, the proximal joint can constrain the position of the distal joint.

**Figure 3:**
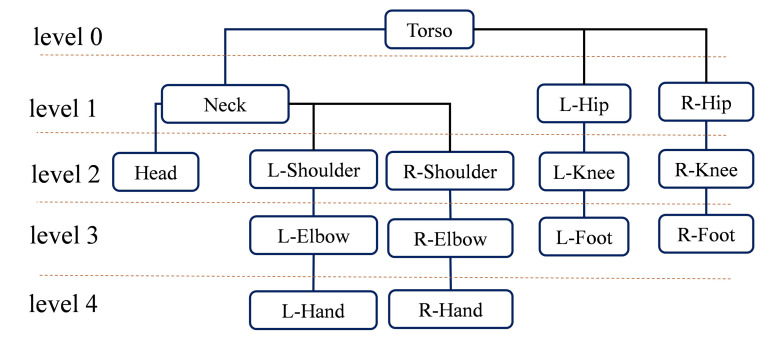
Division of human joints. The level of each joint is denoted on the left side.

In sports rehabilitation, the muscle group surrounding the torso is one of the core muscle groups of the human body. Most motions are performed under the support of the torso muscles. Furthermore, the torso is a vital joint that connects the upper and lower limbs. Based on this, we take the torso joint as the demarcation point and divide both upper limb joints and lower limb joints into different levels, as illustrated in Figure 3. Different joints on the same level share the same number of bones between themselves and the torso. For example, the torso and the left hip are in level one because the number of bones between the neck and torso and that between the left hip and the torso are both one.

It is noted that the data structure ’tree’ can represent the finite nodes with varying levels as a set. In the tree structure, there must be one root node with zero or more direct successor nodes. Except for the root node, other nodes can be partitioned into *n* disjoint finite sets $T_{1},T_{2},\ldots ,T_{n}$. Each finite set can be considered a tree, which is called the subtree of the human-tree. Based on the above analysis, we propose to define the human body as a tree structure called human-tree, as shown in Figure 1b. Nodes in the human-tree correspond to joints of the human body, and connections between nodes correspond to human bones. In particular, leaf nodes in the human-tree correspond to the distal joints of the human body. The depth of each node corresponds to the level of the joint mentioned in Figure 3. As shown in Figure 1b, each branch of the human-tree represents a specific kinematic chain. For example, the branch denoted in black represents the kinematic chain of the right lower limb, and the branch denoted in purple represents the kinematic chain of the right upper limb.

### 3.4. WPL-Based Loss Function

In the tree structure, the weighted path length (WPL) of each leaf node is the product of the weight of the leaf node and the path length from itself to the root node (that is, the depth of the leaf node). The weighted path length (WPL) of the tree is the sum of the weighted path length (WPL) of each leaf node. The WPL of the tree structure can be denoted as:

(3) $$\begin{array}{c} \mathrm{WPL}=\;\sum_{k\in K}w_{k}\cdot L_{k}, \end{array}$$

where $w_{k}$ represents the weight of the leaf node *k*, and $L_{k}$ represents the depth of the leaf node *k*. Because the weight and depth of each node in a given tree remain unchanged, the WPL of the tree remains unchanged. Therefore, WPL can be considered the inherent feature of the tree structure. Similarly, we can also compute the WPL of the human-tree and use the built-in feature to constrain the human pose. This section explains the details of calculating the WPL of the human-tree and global-to-local WPL-based loss functions.

#### 3.4.1. Weight Definition

To compute the WPL of the human-tree, the weight and depth of the distal joint are required. As mentioned in Section 3.2, the depth of the distal joint is the number of bones between the distal joint and the torso. In this section, we explain how to define the weight of the distal joint in our work.

As we all know, the muscle electrical signal carries much information directly relevant to human motion. The muscle electrical signal is employed as the carrier to transmit the motion information to the tendon, which, subsequently, drives the skeleton to complete the motion. Simply, the signal transmission process consists of three steps: (1) the source sends the signal, (2) the channel transmits the signal, and (3) the sink receives the signal. If the channel length is long in a non-ideal environment, the signal is more likely to be distorted.

Similarly, joints can be considered sources or sinks, and bones can be considered channels in the human body. For example, as shown in Figure 1b, for the branch from the torso to the left hand, the torso node is considered the source node, and other nodes are considered the sink nodes. With the increase in the bone length between the sink node and the source node, the signal is more likely to be interfered with, and the quality of the signal becomes worse. The less useful information is transmitted, the less the sink node is controlled by the source node. All in all, the degree of control by the source node to the sink node is inversely proportional to the bone length between the two nodes, and we define the degree of control as the weight of the sink node. Specifically, the weight of leaf node *j* in the human-tree can be denoted as:

(4) $$\begin{array}{c} C_{j}=\sum_{n=1}^{N}L_{n}, \end{array}$$

(5) $$\begin{array}{c} {\tilde{W}}_{j}=\frac{1}{C_{j}}, \end{array}$$

(6) $$\begin{array}{c} W_{j}=\frac{e^{{\tilde{W}}_{j}}}{\sum_{j\in J}e^{{\tilde{W}}_{j}}}, \end{array}$$

where the number of bones between the leaf node *j* and the root node is *N*, $L_{n}$ represents the length of the *n*-th bone, *J* represents the set of all distal joints, $C_{j}$ represents the sum of the *N* lengths of bones, and $W_{j}$ represents the normalized weight of leaf node *j*, which can be acquired using the softmax function.

Of course, there are some methods that use graph structure [42] to model the human body. They consider the human body as a graph and use GCN to extract the human pose features. Specifically, each joint is regarded as the node of the graph, and each bone is considered the edge of the graph. When employing GCN, there is also a concept of weight in the process of feature extraction. Different from the weight of the leaf node defined in our work, the weight in GCN is dependent on the adjacency matrix of the graph. The node with a larger degree contains less useful information and is assigned a smaller weight. This weight definition ignores that the bone length between the two joints affects the quality of information transmission.

#### 3.4.2. Calculation of WPL-Based Loss Function

As mentioned in Section 3.3, WPL is a built-in feature of the human-tree, which can be used to constrain the human pose. In this section, we explain the details of calculating the WPL-based loss function.

For the same subject, no matter his (her) pose changes, the length and the number of the bones are unchanged. Therefore, the WPL of the tree structure is unchanged. Based on this, we can calculate the WPL-based loss between the estimated and the ground-truth joints. For the *b*-th sample on the ITOP dataset, the WPL-based loss is denoted as:

(7) $$\begin{array}{c} l_{b}=\left|\right|H_{b}-{\hat{H}}_{b}\left|\right|, \end{array}$$

where $H_{b}$ and ${\hat{H}}_{b}$ represent the ground-truth and estimated WPL of *b*-th sample, $\left||.|\right|$ represents the L1 loss function. WPL is calculated in the way illustrated in Equation (3). We explain the calculation process of the ground-truth WPL $H_{b}$ in detail. As shown in Figure 1b, there are five leaf nodes (distal joints). We take the L-hand node as an example to illustrate the calculation process of the weight path length. There are four bones between the L-hand node and the torso node, so the path of the L-hand is four. The length of the bone between node *u* and node *v* can be calculated in the following way:

(8) $$L_{\mathrm{uv}}=\sqrt{{\left(x_{u}-x_{v}\right)}^{2}+{\left(y_{u}-y_{v}\right)}^{2}+{\left(z_{u}-z_{v}\right)}^{2}},$$

where $L_{\mathrm{uv}}$ represents the length of the bone between node *u* and node *v*, $\left(x_{u},\;y_{u},z_{u}\right)$ represents the 3D coordinates of node *u*, and $\left(x_{v},\;y_{v},z_{v}\right)$ represents the 3D coordinates of node *v*. It is noted that node *v* and node *u* are connected by only one bone. Then, the sum of the lengths of the above four bones $Y_{\mathrm{lhand}}$ can be denoted as:

(9) $$Y_{\mathrm{lhand}}=\sum L_{\mathrm{uv}},$$

The weight of the L-hand ${\tilde{W}}_{\mathrm{lhand}}$ can be calculated as:

(10) $${\tilde{W}}_{\mathrm{lhand}}=\frac{1}{Y_{\mathrm{lhand}}},$$

Then, the normalized weight of the L-hand can be denoted as:

(11) $$W_{\mathrm{lhand}}=\frac{e^{{\tilde{W}}_{\mathrm{lhand}}}}{e^{{\tilde{W}}_{\mathrm{lhand}}}+e^{{\tilde{W}}_{\mathrm{rhand}}}+e^{{\tilde{W}}_{\mathrm{lfoot}}}+e^{{\tilde{W}}_{\mathrm{rfoot}}}+e^{{\tilde{W}}_{\mathrm{neck}}}},$$

where ${\tilde{W}}_{\mathrm{rhand}}$ represents the weight of the r-hand, ${\tilde{W}}_{\mathrm{rfoot}}$ represents the weight of the l-foot, ${\tilde{W}}_{\mathrm{rfoot}}$ represents the weight of the r-foot, and ${\tilde{W}}_{\mathrm{neck}}$ represents the weight of the neck. Finally, the weighted path length of the L-hand ${\mathrm{WPL}}_{\mathrm{lhand}}$ can be calculated as:

(12) $${\mathrm{WPL}}_{\mathrm{lhand}}=W_{\mathrm{lhand}}\cdot 4,$$

The weighted path length of other leaf nodes can be acquired in the above way.

### 3.5. Global-to-Local WPL-Based Loss Function

**Figure 4:**
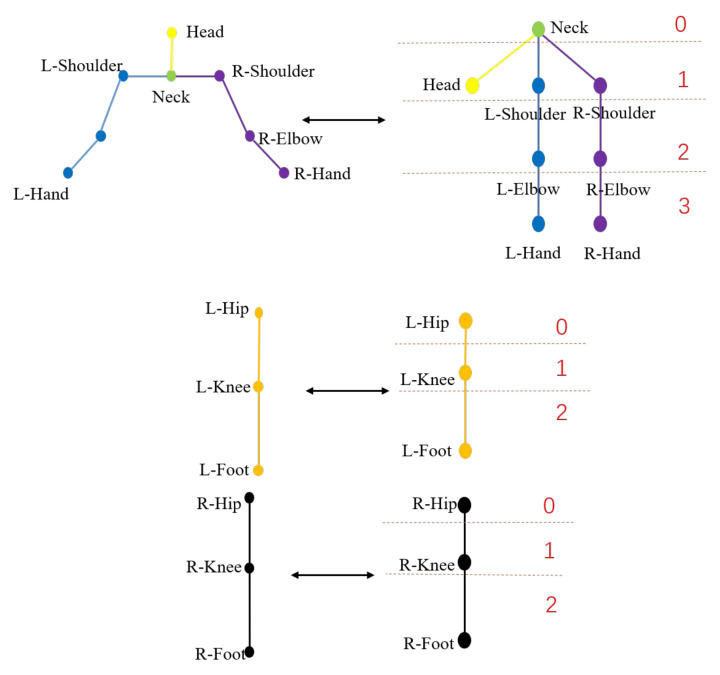
The subtrees and the corresponding local kinematic chains.

The kinematic chain can be divided into two types: the global kinematic chain and the local kinematic chain. As shown in Figure 1, we define the branch starting from the torso as the global kinematic chain, and the branch not starting from the torso as the local kinematic chain. For example, the branch that begins at the torso and terminates at the right hand is defined as the global kinematic chain. In fact, some local human motions are implemented by the local kinematic chain. For example, we only need the local kinematic chain that begins at the right shoulder and ends at the right hand to rotate the right hand. If we only use the global human-tree shown in Figure 1b to constrain the human pose, the local motion information is ignored. It is noted that the local kinematic chain can be considered the subtree of the human-tree. We show some examples of the subtrees and the corresponding local kinematic chains in Figure 4. For example, at the top of Figure 4, we show the subtree constructed by the upper limbs. At this time, the neck joint becomes the root node, and each branch represents the kinematic chain where the first proximal joint is the neck rather than the torso. Similarly, we can calculate the WPL of the subtree in the manner described above. We define the WPL of the human-tree as the global WPL and the WPL of the subtree as the local WPL. Similarly, we can calculate the WPL of the subtree in the way mentioned above. Then we can use both the global and local WPLs to constrain the human pose. For the *b*-th sample, the global-to-local WPL of the human-tree $G_{b}$ can be denoted as:

(13) $$\begin{array}{c} G_{b}=\sum_{q=0}^{Q}P_{q}, \end{array}$$

where *Q* is the number of the subtree, $P_{0}$ represents the WPL of the global tree, and $P_{q}$ represents the WPL of the *q*-th subtree. Then, the global-to-local WPL-based loss function can be denoted as:

(14) $$\begin{array}{c} \mathrm{loss}=\;\sum_{b=1}^{B}\left||{\mathrm{WPL}}_{b}-{\hat{\mathrm{WPL}}}_{b}|\right|, \end{array}$$

where ${\hat{\mathrm{WPL}}}_{b}$ and ${\mathrm{WPL}}_{b}$ represent the estimated and ground-truth global-to-local WPL of the *b*-th sample, respectively.

It is worth noting that our proposed WPL loss function is well designed according to both the model structure and physical significance of A2J, which is aimed at improving the effectiveness of A2J. The proposed WPL-based loss function is based on the 3D coordinates produced by Equations (1) and (2) and is combined with other loss functions to train A2J. In this way, when the preset anchors estimate the position of a joint, the spatial relationship between joints can be considered from different viewpoints.

### 3.6. End-to-End Learning

Consistent with A2J, we train the baseline framework A2J in an end-to-end manner under the supervision of three loss functions, which include the global-to-local WPL-based loss, informative anchor surrounding loss [27], and joint position estimation loss [27]. The joint position estimation loss function is used to calculate the loss between the estimated and ground-truth 3D coordinates of joints, which can be expressed as:

(15) $${\mathrm{loss}}_{p}=\alpha \sum_{j\in J}L_{\tau 1}\left({\hat{S}}_{j}-T_{j}^{i}\right)+\sum_{j\in J}L_{\tau 2}\left({\hat{D}}_{j}-T_{j}^{d}\right),$$

where α represents the balance factor, and $T_{j}^{i}$ and $T_{j}^{d}$ represent the ground-truth 2D coordinates and depth of joint *j*, respectively. $L_{\tau }\left(.\right)$ represents the L1-smooth loss function, which is denoted as:

(16) $$L_{\tau }\left(x\right)=\left\{\begin{array}{c} \frac{1}{2\tau }x^{2},\;\;\;\;\;\;\mathrm{for}\;\left|x\right|\;<\tau \\ \left|x\right|-\frac{\tau }{2},\;\;\;\;\;\;\;\;\mathrm{otherwise} \end{array}\right.,$$

where, in Equation (15), τ1 is set as 1, and τ2 is set as 3. The informative anchor surrounding loss function is used to make the anchors surrounding the target joints with larger weights, which can be formulated as:

(17) $${\mathrm{loss}}_{p}=\sum_{j\in J}L_{\tau 1}\left(\sum_{a\epsilon A}{\tilde{P}}_{j}\left(a\right)S\left(a\right)-T_{j}^{i}\right),$$

where S(a) represents the 2D coordinates of anchor *a*. Finally, the above two loss functions proposed in A2J are combined with our proposed WPL-based loss function to train the model, which is formulated as:

(18) $${\mathrm{Loss}}_{\mathrm{total}}=\;{\mathrm{loss}}_{\mathrm{WPL}}+\;{\mathrm{loss}}_{a}+{\mathrm{loss}}_{p},$$

where ${\mathrm{Loss}}_{\mathrm{total}}$ represents the loss in all, and ${\mathrm{loss}}_{\mathrm{WPL}}$ represents our proposed loss function.

## 4. Experiments

### 4.1. Dataset and Experimental Setup

**Figure 5:**
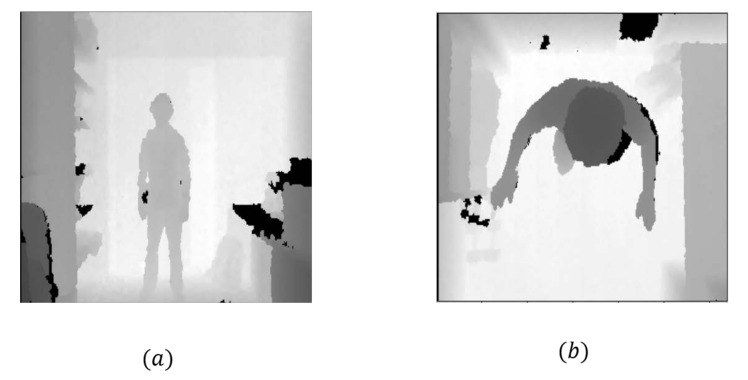
(**a**) ITOP front-view human pose dataset. (**b**) ITOP top-view human pose dataset.

**ITOP front-view human pose dataset.** The ITOP front-view dataset [28] contains 40K training and 10K testing depth images, which are captured from the front view. Each depth image is annotated by 15 joints, as shown in Figure 5a.

**ITOP top-view human pose dataset.** The ITOP top-view dataset [28] contains 40K training and 10K testing depth images, which are captured from the top view. Each depth image is annotated by 15 joints, as shown in Figure 5b.

**Evaluation metric.** We evaluate the performance of our method under the metric of the mean average precision (mAP) with the 10-cm rule [28], which is the average precision of all human body parts. In addition, we present the precision of individual body parts. The mean average precision (mAP) is formulated as:

(19) $$\mathrm{mAP}=\frac{R_{c}}{R},$$

where $R_{c}$ represents the number of successful joints, and *R* represents the number of all the test joints. The predicted joint is successful when the predicted joint is less than 10 cm from the ground-truth in 3D space.

**Model configuration.** We implement our model with Pytorch 1.7 on one GTX-3090Ti GPU. Consistent with A2J [27], data augmentation is also performed in our experiments. We use Adam [43] as the optimizer, and the learning rate is set as 0.00035 with a weight decay of 0.0001.

### 4.2. Comparison with State-of-the-Art Methods

**ITOP front-view human pose dataset.** We compare our method with other methods on the ITOP front-view dataset, and the comparison results are displayed in Table 1. Our method performs well on the ITOP front-view dataset. Since we employ the global-to-local WPL-based constraint on A2J [27], we mainly analyze the comparison results between A2J and our method. The mean accuracy of our method exceeds that of A2J by 0.7%. Specifically, the accuracies of shoulders, elbows, hands, knees, and feet in our method are all higher than those in A2J. These results demonstrate that the proposed global-to-local WPL-based loss can effectively constrain the distal joints of the human body.

**ITOP top-view human pose dataset.** We compare our method with other methods on the ITOP top-view dataset, and the comparison results are shown in Table 2. Our method performs well on the ITOP top-view dataset. Our mean accuracy exceeds that of the A2J method by 0.4%. In particular, the accuracies on elbows and hands in our method are higher than those from A2J, which proves that the proposed global-to-local WPL-based loss can effectively constrain the distal joints.

### 4.3. Ablation Study

We analyze the effectiveness of the global-to-local WPL-based loss on the ITOP front-view dataset. The results of the ablation analyses on the ITOP front-view dataset are displayed in Table 3.

#### 4.3.1. Impact of the Global-to-Local WPL-Based Constraint

In order to validate the effectiveness of the global-to-local WPL-based constraint, we remove both global and local constraints and train the model based on A2J. The experimental results are shown in the second column of Table 3. The mean accuracy and the accuracies of shoulders, elbows, hands, hips, knees, and feet are lower than by using the global-to-local constraint, which proves the effectiveness of the global-to-local constraint.

#### 4.3.2. Impact of the Global WPL-Based Constraint

To validate the effectiveness of the global WPL-based constraint, we remove the global constraint and only use the local constraint. Then we train the model based on A2J. The experimental results are shown in the third column of Table 3. It can be seen that the mean accuracy is higher than that of the method without the global-to-local WPL-based constraint and lower than that of the method with the global-to-local WPL-based constraint, which proves the effectiveness of the global WPL-based constraint.

#### 4.3.3. Impact of the Local WPL-Based Constraint

To validate the effectiveness of the local WPL-based constraint, we remove the local constraint and only use the global constraint. Then we train the model based on A2J. The experimental results are shown in the fourth column of Table 3. It can be seen that the mean accuracy is higher than that of the method without the global-to-local WPL-based constraint and lower than that of the method with the global-to-local WPL-based constraint, which proves the effectiveness of the local WPL-based constraint.

#### 4.3.4. Impact of the Learning Rate and Weight Decay

We also validate our model based on different learning rates with different weight decay on the ITOP top-view dataset. The experimental results are shown in Table 4. It can be seen that our proposed method performs best when the learning rate is set as 0.00035 with a weight decay of 0.0001.

### 4.4. Qualitative Evaluation

**Figure 6:**
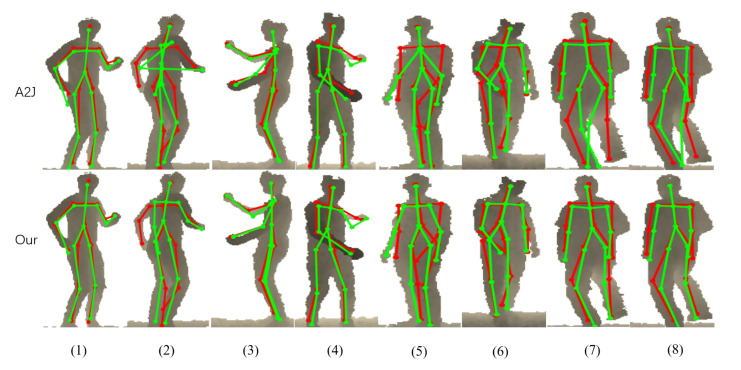
The comparison visualization results with A2J in the 2D plane on the ITOP front dataset. The ground-truth is shown in red, and the estimated result is in green.

**Figure 7:**
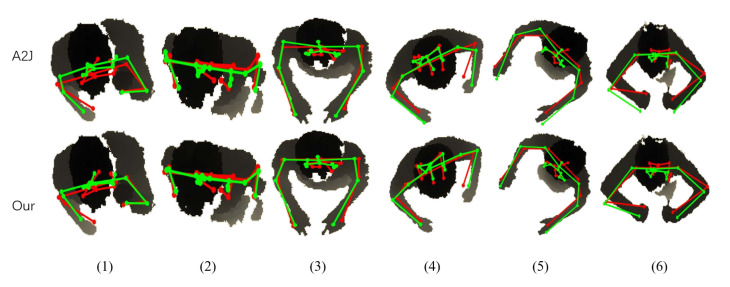
The comparison visualization results with A2J in the 2D plane on the ITOP top dataset. The ground-truth is shown in red, and the estimated result is in green.

We show the comparison visualization results with A2J on the ITOP side and ITOP top datasets in Figure 6 and Figure 7, respectively. The qualitative results show that our method can effectively improve the performance of the distal joints.

As shown in Figure 6, our method can effectively alleviate the phenomenon that the estimated results are far from the ground-truth for distal joints. For columns (7) and (8) in Figure 6, the estimated results of the lower limbs in our method are closer to the ground-truth compared to A2J. Our method also performs well on the upper limbs. For example, the sharp offsets from the ground-truth of the shoulders lead to it being hard for the estimated results to be seen as a human body. Although the estimated results in our method are not fully equal to the ground-truth, they can be clearly seen as a human body.

Figure 7 also validates that our method can effectively improve the performance of the distal joints. Specifically, the estimated results of hands are improved in columns (4)–(6), and the estimated results of the shoulders are improved in columns (1)–(3).

## 5. Conclusions

In this paper, we model the human skeleton as the human-tree and propose a global-to-local WPL-based loss function. The proposed loss function can constrain the distal joint with all the proximal joints on the same kinematic chain. The experimental results validate that our method can improve the accuracy of the distal joints on two human pose datasets. In the future, we will impose our proposed WPL-based loss function on other baseline models to further validate the effectiveness of our proposed method.

## Figures and Tables

**Table 1:** Performance comparison on the ITOP front-view dataset. We evaluate the methods that use the metric of the mean average precision (mAP) with the 10-cm rule. We show the results of each human joint and the average results of all the human joints.

Method	RF [24]	IEF [44]	MatchNet [17]	VI [28]	RTW [23]	CMB [33]	REN-9x6x6 [45]	V2V [26]	A2J [27]	Ours
Head	63.8	96.2	95.6	98.1	97.8	97.7	98.7	98.29	98.54	98.46
Neck	86.4	85.2	94.2	97.5	95.8	98.5	99.4	99.07	99.20	99.11
Shoulders	83.3	77.2	87.3	96.5	94.1	75.9	96.1	97.18	96.23	97.14
Elbows	73.2	45.4	72.5	73.3	77.9	62.7	74.7	80.42	78.92	80.10
Hands	51.3	30.9	53.8	68.7	70.5	84.4	55.2	67.26	68.53	**69.19**
Torso	65.0	84.7	85.4	85.6	93.8	96.0	98.7	98.73	98.52	98.52
Hips	50.8	83.5	70.5	72.0	90.3	87.9	91.8	93.23	90.85	92.27
Knees	65.7	81.8	64.2	69.0	68.8	84.4	89.0	91.80	90.75	91.39
Feet	61.3	80.9	58.8	60.8	68.4	83.8	81.1	87.60	86.91	86.98
Mean	65.8	71.0	72.62	77.4	80.5	83.3	84.9	**88.7**	88.0	**88.7**

**Table 2:** Performance comparison on the ITOP top-view dataset. We evaluate the methods that use the metric of the mean average precision (mAP) with the 10-cm rule. We show the results of each human joint and the average results of all the human joints.

Method	RF [24]	IEF [44]	RTW [23]	VI [28]	REN-9x6x6 [45]	A2J [27]	Ours
Head	95.4	83.8	98.4	98.1	98.2	98.38	98.19
Neck	98.5	50.0	82.2	97.6	98.9	98.91	98.66
Shoulders	89.0	67.3	91.8	96.1	96.6	96.26	95.89
Elbows	57.4	40.2	80.1	86.2	74.4	75.88	75.78
Hands	49.1	39.0	76.9	85.5	50.7	59.35	**61.72**
Torso	80.5	30.5	68.2	72.9	98.1	97.82	97.78
Hips	20.0	38.9	55.7	61.2	85.5	86.88	86.63
Knees	2.6	54.0	53.9	51.6	70.0	79.66	79.27
Feet	0.0	62.4	28.7	51.5	41.6	58.34	**60.5**
Mean	47.4	51.2	68.2	75.4	75.5	80.5	**80.9**

**Table 3:** The ablation results on the ITOP front-view dataset.

	w/o Global and Local Constraint	w/o Global Constraint	w/o Local Constraint	Ours
Head	98.54	98.56	98.58	98.46
Neck	99.20	99.12	99.18	99.11
Shoulders	96.23	96.78	96.79	**97.14**
Elbows	78.92	80.15	79.89	80.10
Hands	68.53	69.10	69.12	**69.19**
Torso	98.52	98.97	98.97	98.52
Hips	90.85	92.02	92.25	**92.27**
Knees	90.75	90.63	90.94	**91.39**
Feet	86.91	84.60	84.77	**86.98**
Mean	88.0	88.2	88.3	**88.7**

**Table 4:** Impact of the learning rate and weight decay on the ITOP top-view dataset.

	Weight Decay = 0.0001	Weight Decay = 0.0002
learning rate = 0.00025	80.6	80.7
learning rate = 0.00035	**80.9**	80.8
learning rate = 0.00045	80.4	80.6
